# Single-cell sequencing reveals the tumor immune microenvironment in thyroid cancer: a narrow review

**DOI:** 10.3389/fimmu.2025.1738583

**Published:** 2026-01-05

**Authors:** Kangcong Liang, Ziyu Wang, Zhiqiang Zhang, Gengluan Liu, Xidi Wang, Heng Cao, Ming Zhong, Liping Ye, Xin Zhong, Jingyu Xun, Kefeng Lei, Ningning Li

**Affiliations:** 1 Tomas Lindahl Nobel Laureate Laboratory, The Seventh Affiliated Hospital of Sun Yat-sen University, Shenzhen, Guangdong, China; 2 Department of General Surgery, The Seventh Affiliated Hospital of Sun Yat-Sen University, Shenzhen, Guangdong, China; 3 Digestive Diseases Center, Guangdong Provincial Key Laboratory of Digestive Cancer Research, The Seventh Affiliated Hospital of Sun Yat-sen University, Shenzhen, Guangdong, China; 4 Respiratory Medicine, The Seventh Afffliated Hospital of Sun Yat-Sen University, Shenzhen, Guangdong, China; 5 Scientific Research Center, The Seventh Affiliated Hospital of Sun Yat-Sen University, Shenzhen, China; 6 Future Medical Center, Shenzhen University of Advanced Technology, Shenzhen, Guangdong, China

**Keywords:** thyroid neoplasms, tumor microenvironment, single-cell gene expression analysis, immune, precision immunotherapy

## Abstract

The immune microenvironment profoundly shapes the progression and therapeutic response of thyroid carcinoma. Through comprehensive analysis of single-cell RNA sequencing data, this review delineates the immune landscapes of papillary thyroid carcinoma (PTC), poorly differentiated thyroid carcinomas (PDTC), and anaplastic thyroid carcinomas (ATC), revealing a differentiation dependent trajectory of tumor immune microenvironment remodeling—from immune activation suppression coexistence in PTC, to immune exclusion in PDTC, and terminal exhaustion in ATC. This single-cell based approach enables high resolution dissection of cellular heterogeneity, immune crosstalk, and spatial organization that are often masked in bulk analyses. Such insights provide a scientific basis for precision immunotherapy, offering guidance for differentiation tailored strategies to overcome immune escape and improve clinical outcomes in thyroid cancer.

## Graphical Abstract

**Figure:**
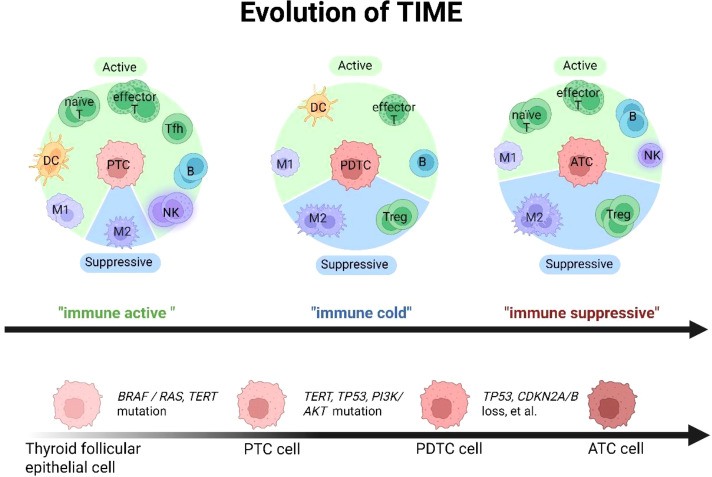

## 1 Introduction

Thyroid carcinoma (TC) is one of the most common malignant tumors of the endocrine system, and its biological behavior, therapeutic response, and prognosis are closely associated with the degree of tumor differentiation (1). According to histological differentiation, TC is generally classified into differentiated types—differentiated thyroid carcinoma (DTC, such as papillary thyroid carcinoma (PTC) and follicular thyroid carcinoma (FTC)), poorly differentiated thyroid carcinoma (PDTC) and anaplastic thyroid carcinoma (ATC) (2). Clinically, DTC usually exhibits slow progression and favorable prognosis, whereas PDTC and ATC are characterized by high proliferative activity, extensive genetic abnormalities, and resistance to therapy, resulting in a poor clinical outcome (3, 4).

The prognosis of tumors largely depends on their biological behavior, which is profoundly influenced by the tumor immune microenvironment (TIME) (5). As a dynamic ecosystem composed of immune cells, stromal cells, cytokines, and metabolic products, the TIME plays a pivotal role in regulating the initiation, progression, and therapeutic sensitivity of TC (6–8). The immune composition and functional state of the TIME not only determine the invasiveness and metastatic potential of tumor cells but also affect patients’ clinical outcomes and treatment responses (9). Based on differentiation gradients, single-cell RNA sequencing (scRNA-seq) has systematically delineated distinct immune cell subpopulations and their functional states within the TIME, revealing the immunoregulatory landscape across different differentiation stages of TC (10, 11).

Dissecting this complexity requires advanced transcriptional profiling tools. While bulk RNA sequencing has been instrumental in identifying overall immune signatures, it obscures the cellular heterogeneity central to the function of TIME. In contrast, scRNA-seq resolves this diversity by cataloging immune and stromal subpopulations at unprecedented resolution, directly revealing their functional states and communication networks. Furthermore, spatial transcriptomics is now critical for mapping these cellular interactions within their native tissue architecture, validating the ecological niches predicted by scRNA-seq. A comparative overview of these technologies is provided in **Table 1**.

**Table 1:** Comparative overview of key transcriptomic profiling technologies for TIME analysis.

Technologies	Principles	Advantages	Limitations
Bulk RNA sequencing	Measures average gene expression from a tissue lysate	Cost-effective for identifying cohort-level immune gene signatures	Obscures cellular heterogeneity and rare but functionally critical populations
scRNA-seq	Profiles transcriptomes of individual cells	Uncovers cellular diversity, novel cell states, and cell-cell communication networks	Loses native tissue architecture and spatial context
Spatial Transcriptomics	Maps gene expression within its original tissue location	Preserves spatial context, visualizing ecological niches (e.g., immune excluded)	Lower cellular resolution; data spots often contain multiple cells

scRNA-seq, single-cell RNA sequencing.

Studies have demonstrated that, along with the loss of differentiation in TC, the TIME exhibits a progressive transition from a state of “coexisting activation and suppression” (an inflamed microenvironment with a delicate balance of effector and suppressor forces) to “low infiltration/exclusion” (a paucimmune landscape lacking productive infiltration) and eventually to “high infiltration with exhaustion” (a high-density but dysfunctional state dominated by T-cell exhaustion and myeloid-driven suppression) (12–14). In ATC, such immune exhaustion is frequently characterized by CD8^+^ T cells dysfunction and enrichment of regulatory T cells (Tregs) (15). A summary of representative studies describing the immune composition and major findings across differentiation types is presented in **Table 2**.

**Table 2:** Recent studies on TIME in thyroid cancer.

Types	Samples (n)	Tissue	Ages	Stages	Mutations	Major findings	References
PTC	10	Fresh	NA	6 T1 4 T2/N1b	*RAS/BRAF*	TIL-B cells are enriched, GC-B cells are selectively enriched, T cells are abundant; GC-B cells actively proliferate and support TLSs formation, aiding immune balance and tumor control.	Li C, et al., 2025 (16)
PTC	6	Fresh	NA	NA	*BRAF^V600E^*	In metastases, B and NK cells increased, T and myeloid cells decreased, reflecting immune shifts with metastatic status; infiltration remained abundant but heterogeneous.	Zheng G, et al., 2025 (17)
PDTC	14	FFPE	NA	NA	5 *RAS/TERT* 9 WT	The tumor showed lowest TIL scores, with CD8^+^ T cells decreased yet relatively enriched but functionally inactive, exhausted CD8^+^ T cells increased, alongside marked reductions in TAMs and B cells.	Giannini R, et al., 2019 (18)
PDTC	28	FFPE	17-77	NA	NA	The tumor ecosystem exhibits low levels of CD8^+^ T cells, TAMs, neutrophils and DCs, leading to sparse infiltration, weak immunosuppression and minimal signaling that limits tumor clearance without potent immune evasion.	Pan Z, et al., 2025 (19)
ATC	5	Fresh	NA	NA	NA	T cells are reduced and exhausted, B cells decreased and dysfunctional, and TAMs skewed to M2 polarization—collectively fostering an immunosuppressive milieu that drives immune evasion and drug resistance.	Pan Z, et al., 2022 (20)
ATC	20	Fresh	NA	NA	NA	Increased TAMs with M2 enrichment drive tumor progression through crosstalk with ATC stem cells, thereby remodeling the TIME.	Liu Q, et al., 2025 (21)

Single-cell and bulk profiling reveal a differentiation dependent evolution of the thyroid cancer immune microenvironment, from TLSs forming B cells activation in PTC to immune depleted and functionally suppressed ecosystems in PDTC and ATC, driven by exhaustion, M2 polarized TAMs, and disrupted lymphoid myeloid balance. TLSs, tertiary lymphoid structures; TIL, Tumor Infiltrating Lymphocytes score; GC-B cell, Germinal Center B cell; TAMs, Tumor-Associated Macrophages; DCs, Dendritic cells; M2, M2 type Tumor-Associated Macrophage; TIME, tumor immune microenvironment; PTC, papillary thyroid carcinoma; PDTC, poorly differentiated thyroid carcinoma; ATC, anaplastic thyroid carcinoma; FFPE, formalin-fixed/paraffin-embedded; NA, Not Available.terms. After removing duplicates, 653 records were screened, yielding 134 studies relevant to this review. The literature comprised a core of 27 studies featuring transcriptomics sequencing data, complemented by a collection of supporting articles. These supporting articles were further subdivided into four categories: 15 reviews, 36 studies without single-cell data, 27 clinical studies, and 29 supplementary references.

The immune heterogeneity across different differentiation states provides new insights for clinical management. First, immune profiling can serve as an auxiliary reference for pathological classification and risk assessment, identifying specific immune subpopulations may help infer the degree of tumor differentiation and potential aggressiveness (13). Second, immunosuppressive TIME signatures are associated with unfavorable clinical outcomes, including increased risks of recurrence and resistance to radioactive iodine therapy (22, 23). Third, persistent activation of immune checkpoint pathways in patients with PDTC and ATC provides a biological rationale for the application of PD-1/PD-L1 and CTLA-4 inhibitors. Meanwhile, targeting intercellular communication networks represents a promising strategy for future combinational “immunotherapy + targeted therapy” approaches (24, 25).

On this basis, this review centers on the axis of “differentiation gradient–TIME heterogeneity–clinical translation”, systematically summarizing the major immune landscape features of thyroid carcinoma and their potential clinical implications, thereby providing a solid theoretical and practical foundation for precision diagnosis, risk stratification, and personalized immunotherapy in TC.

## 2 Methods and search strategy

**Figure 1:**
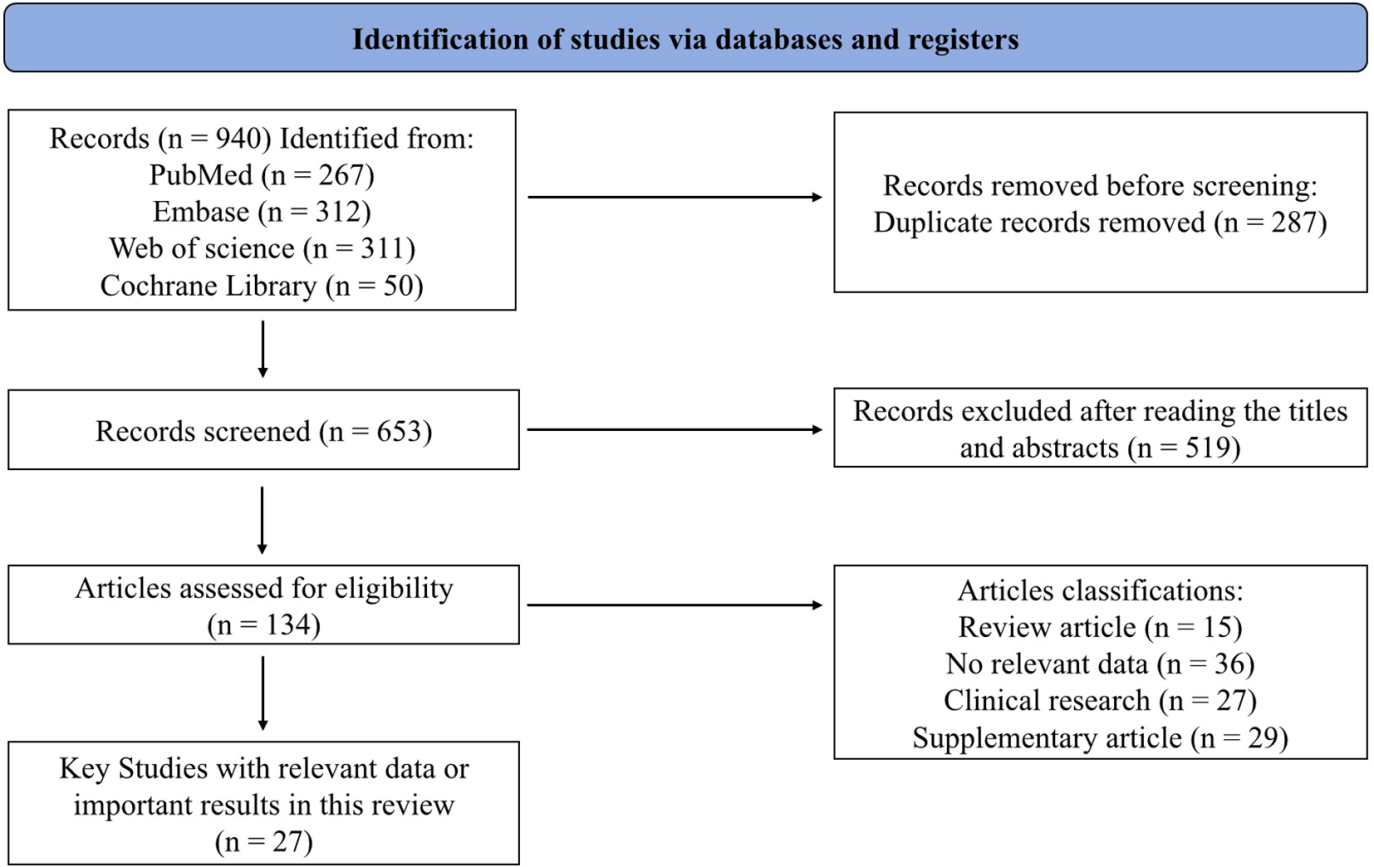
Flow diagram illustrating the identification, screening, eligibility assessment, and inclusion of studies in this review. 940 records from PubMed, Embase, Web of Science, and Cochrane Library based on predefined MeSH terms. Removing 653 duplicated records, yielding 134 studies relevant to this review. The 134 articles comprised a core of 27 studies with transcriptomics data and supporting literatures, the latter further divided into 15 reviews, 36 studies without single-cell data, 27 clinical studies, and 29 supplementary references.

To ensure a comprehensive and unbiased retrieval of relevant literature, a systematic search was conducted across four major electronic databases: PubMed, Web of Science, Embase and Cochrane Library. The search strategy employed the following keywords and their combinations: “Thyroid Neoplasms”, “Thyroid Cancer, Papillary”, “Adenocarcinoma, Follicular”, “Thyroid Carcinoma, Anaplastic”, “Single-Cell Gene Expression Analysis”, “Tumor Microenvironment”, “Tumor-Associated Macrophages”, “Dendritic Cells”, “Killer Cells, Natural” to retrieve English articles published from January 2015 to October 2025. The final literatures included for in-depth analysis were selected based on strict criteria: (1) high relevance to the research theme; (2) rigorous experimental design; (3) complete and reliable data. To ensure objectivity, a double-blind screening process was adopted, where two researchers independently evaluated literature quality, with discrepancies resolved through expert group discussions. In the comprehensive analysis, we paid special attention to the mutual verification between different studies, incorporating both supporting evidence and opposing viewpoints to ensure the scientificity and reliability of conclusions. The overall literature screening and inclusion process is summarized in **Figure 1**. 940 records were retrieved from PubMed, Embase, Web of Science, and Cochrane Library based on predefined MeSH.

### 2.1 Technical considerations for scRNA-seq platforms

Among the scRNA-seq datasets included in this review, most were generated using the 10x Genomics Chromium system, with fewer studies using BD Rhapsody. These platforms differ in throughput, sensitivity, and suitability for thyroid tissue. The 10x system enables high-throughput profiling of heterogeneous tumors, whereas BD Rhapsody provides greater molecular sensitivity but lower cell capacity. In addition to platform-specific features, several technical limitations should be acknowledged, including batch effects, differences in gene-detection sensitivity and clustering performance across platforms, and biases in immune-cell abundance estimation arising from variable tumor-to-immune cell ratios. Biological and clinical sources of variability also influence data interpretation, such as heterogeneity between primary and metastatic sites, pre- versus post-treatment sampling, and potential inaccuracies introduced by marker-based versus automated annotation strategies. These factors collectively highlight the need for caution when comparing scRNA-seq results across studies.

## 3 TIME of DTC

DTC primarily includes PTC and FTC, accounts for more than 90% of all malignant thyroid tumors (1), with a subset of patients progressing to radioiodine-refractory disease (26). In recent years, beyond the classical driver mutations in *BRAF*, *RAS*, and *TERT* (27), increasing attention has been directed toward the pivotal role of TIME in the initiation, progression, and therapeutic response of PTC (28, 29).

**Figure 2:**
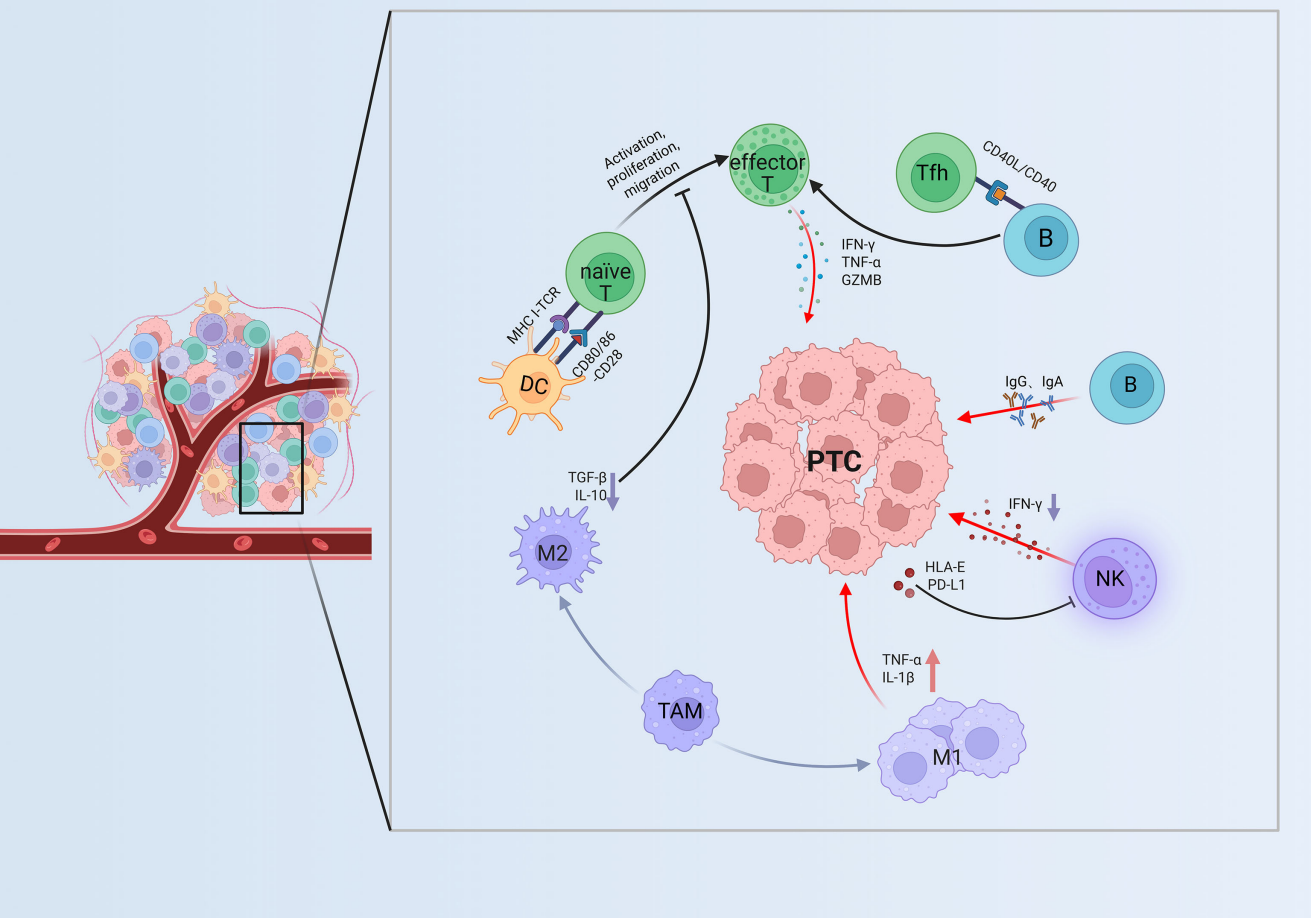
Immune interactions in the TIME of PTC. DCs activate naïve T cells through MHC–TCR and CD80/CD86–CD28 signaling, generating effector T cells that secrete IFN-γ, TNF-α, and GZMB for tumor killing. Tfh cells and B cells via CD40/CD40L binding to support T cell immunity, while NK cells contribute cytotoxicity. M1 macrophages release pro-inflammatory factors, and M2 macrophages produce TGF-β and IL-10, forming a balanced immune active TIME. ↑Purple downward arrows indicate decreased expression; ↓red upward arrows indicate increased expression. DC, Dendritic cell; naïve T, naïve T cell; effector T, effector T cell; Tfh, T follicular helper cell; B, B cell; NK, NK cell; M1, M1 type Tumor-Associated Macrophage; M2, M2 type Tumor-Associated Macrophage; TAM, Tumor-Associated Macrophages; PTC, papillary thyroid carcinoma; TIME, tumor immune microenvironment. GZMB, Granzyme B; HLA-E, Human Leukocyte Antigen–E; MHC-I, Major Histocompatibility Complex Class I; TCR, T Cell Receptor.

Given that PTC represents the predominant subtype of DTC, this review focuses on the immunological landscape of DTC using PTC as a representative model. Histologically, PTC tissues are characterized by extensive infiltration of macrophages, T cells, natural killer (NK) cells, neutrophils, and dendritic cells (DCs) (11, 29). ScRNA-seq studies have revealed that the TIME of PTC exhibits a state of “coexisting immune activation and suppression”: on one hand, active antitumor immune cell populations are present, while on the other hand, phenomena such as T cells exhaustion, enrichment of immunosuppressive macrophages, and impaired antigen presentation are frequently observed (11, 18). The complex cellular interactions within the TIME of PTC are illustrated in **Figure 2**, highlighting the interplay among T cells, B cells, NK cells, DCs, and macrophage subpopulations.

### 3.1 T cells: immune activation and exhaustion

T cells constitute the predominant immune population, accounting for approximately 40%–60% of immune cells (30, 31). scRNA-seq analyses reveal distinct lineage differentiation within the T cells compartment, including CD8^+^ cytotoxic T cells, CD4^+^ helper T cells (Th1, Th2, and Tfh), and Tregs (32). CD8^+^ T cells exhibit high expression of cytotoxic molecules such as PRF1 (Perforin-1), GZMB (Granzyme B), and IFN-γ, along with upregulation of exhaustion markers PD-1, HAVCR2 (TIM-3), and LAG3, indicating functional impairment under persistent antigen stimulation (33). Among CD4^+^ T cells, the Th1 (TBX21^+^) and Tfh (CXCL13^+^, IL21^+^) subsets are predominant. Th1 cells promote antitumor immunity through IFN-γ signaling, while Tfh cells form a cooperative immune axis with B cells via the CD40L–CD40 and IL-21–IL-21R pathways (34, 35). The proportion of FOXP3^+^ Tregs is elevated in highly aggressive PTC, where they suppress effector T cell activity through secretion of TGF-β and IL-10 (36). Cell–cell interaction analyses further demonstrate that T cells, macrophages, and dendritic cells communicate through regulatory axes such as PD-L1/PD-1 and CD80/CD28, while Tregs and plasmacytoid dendritic cells cooperatively contribute to tumor immune evasion (7, 36).

### 3.2 Macrophages: remodeling immune homeostasis

Tumor-associated macrophages (TAMs) constitute approximately 20%–30% of immune cells in PTC (7, 37). Two major macrophage subtypes have been identified: proinflammatory M1-like (CXCL9^+^, IL1B^+^) and immunosuppressive M2-like (CD163^+^, MRC1^+^) populations (11, 35). In early stage or low risk PTC, M1-type macrophages are more prevalent and secrete cytokines such as IL-1β and TNF-α to promote immune mediated tumor clearance (38). In contrast, M2 type TAMs are significantly enriched in advanced or *BRAF*-mutant PTC, producing IL-10, CCL18, and TGF-β, which facilitate epithelial–mesenchymal transition and angiogenesis (39, 40). Moreover, TAMs recruit Tres via the CCL22–CCR4 signaling axis, thereby establishing and reinforcing an immunosuppressive network within the tumor microenvironment (41).

### 3.3 NK cells: polarization and suppression

Although NK cells represent a minor population, accounting for approximately 5%–10% of immune cells, their functional state plays a crucial role in antitumor defense (31). NK cells can be broadly categorized into two subtypes: activated (NKG7^+^, PRF1^+^, GZMB^+^) and inhibitory (KLRC1^+^, TIGIT^+^, LAG3^+^) phenotypes (42). In progressive PTC, inhibitory NK cells are markedly enriched, characterized by the downregulation of cytotoxic genes and reduced IFN-γ secretion (43). Tumor cells suppress NK cells cytotoxicity through upregulation of HLA-E and PD-L1, which interact with inhibitory receptors NKG2A and PD-1 on NK cells (31, 39). In addition, disruption of the IL-15–IL-15R signaling axis limits NK cells maturation and contributes to their functional impairment (44).

### 3.4 B cells: activation and immunosuppressive reprogramming

B cells in PTC exhibit dual characteristics of both immune activation and suppression (45). Data reveal that the B cells compartment comprises naïve (MS4A1^+^), activated (CD27^+^), and plasmablast-like (XBP1^+^, MZB1^+^) populations (16). Activated B cells enhance Tfh cells function through antigen presentation and the CD40–CD40L signaling pathway, thereby forming a supportive immune axis (46). Plasmablast-like B cells secrete immunoglobulins such as IgG and IgA, mediating antitumor responses or promoting immune inflammation (31, 45). A subset of B cells also upregulates IL-10 and PD-1 expression, exhibiting immunosuppressive properties (47).

### 3.5 DCs: antigen presentation and spatial localization

DCs constitute a relatively small fraction of immune cells in PTC but play a pivotal role in initiating immune responses (36). Two major DCs subsets—cDC1 and cDC2—have been identified in PTC (33). In early stage disease, cDC1s exhibit robust antigen presenting capacity, characterized by high expression of HLA-DRA, CD80, and CD86, effectively activating CD8^+^ T cells (48). However, in advanced or immunosuppressive contexts, the proportion of cDC1s decreases, IL-12 signaling is attenuated, and PD-L1 expression is elevated, leading to impaired antigen presentation (33, 48). cDC2s primarily secrete IL-10 and CCL17, which facilitate Tregs recruitment (49). Spatial transcriptomics analyses reveal that DCs are predominantly located at the tumor periphery, forming an “immune activation zone” in conjunction with T cells, suggesting this region may represent an immunotherapy responsive niche (33).

## 4 TIME of PDTC

**Figure 3:**
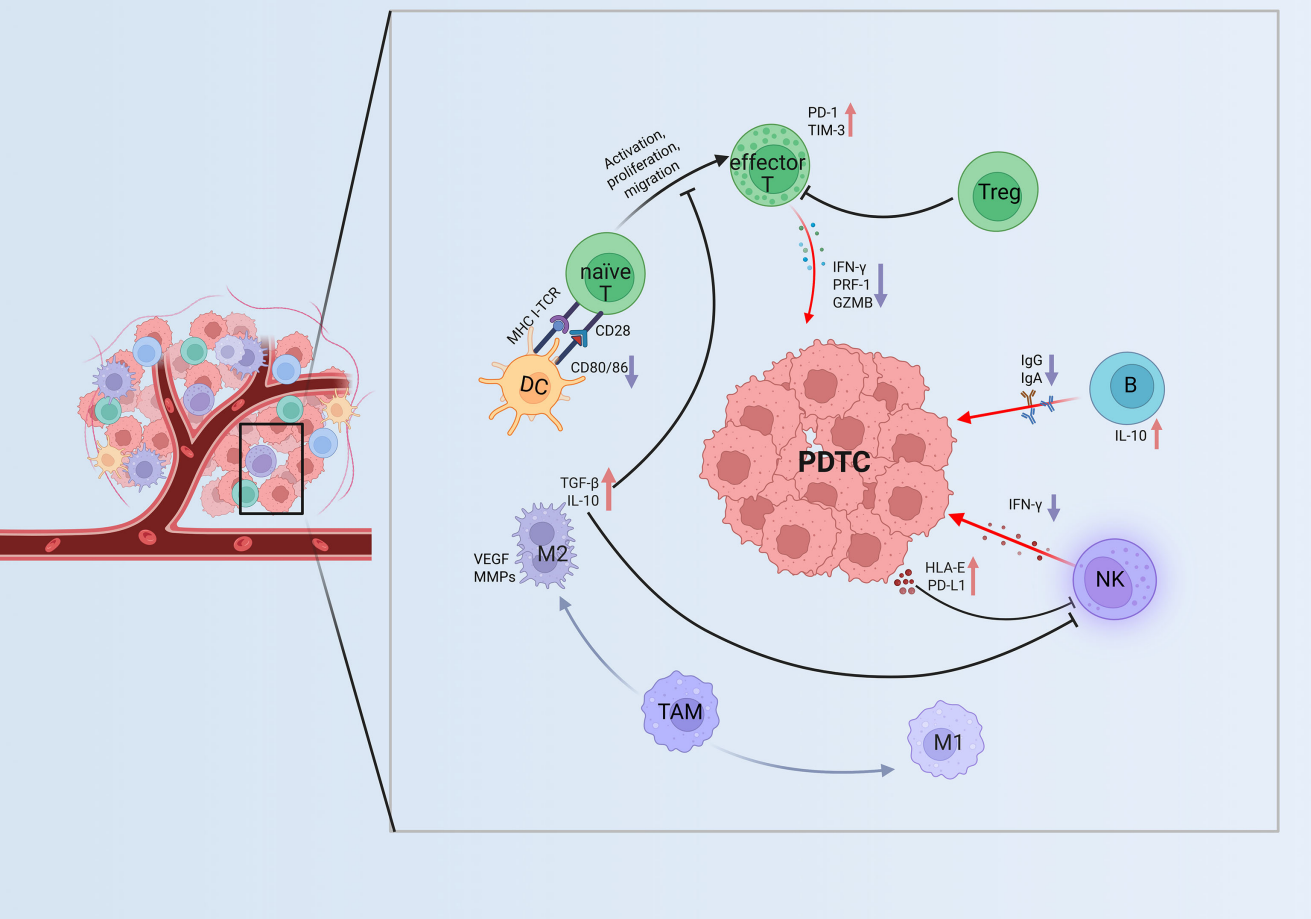
Immunoregulation within the TIME of PDTC. Effector T cells upregulate PD-1 and TIM-3 and reduce PRF-1 and GZMB expression, showing partial exhaustion. Tregs and M2 TAMs enriched in TGF-β, IL-10, and VEGF suppress effector activity and promote remodeling. NK and B cells exhibit impaired cytotoxicity and antibody production. These interactions indicate a shift toward an immunosuppressed TIME in PDTC. ↑Purple downward arrows indicate decreased expression; ↓red upward arrows indicate increased expression. PDTC, poorly differentiated thyroid carcinoma; DC, Dendritic cell; naïve T, naïve T cell; effector T, effector T cell; Treg, Regulatory T cell; B, B cell; NK, NK cell; M1, M1 type Tumor-Associated Macrophage; M2, M2 type Tumor-Associated Macrophage; TAM, Tumor-Associated Macrophages; TIME, tumor immune microenvironment; GZMB, Granzyme B; PRF-1, Perforin-1; HLA-E, Human Leukocyte Antigen–E; MHC-I, Major Histocompatibility Complex Class I; TCR, T Cell Receptor; VEGF, Vascular Endothelial Growth Factor; MMPs, Matrix Metalloproteinases.

PDTC, positioned between PTC/FTC and ATC, represents an intermediately aggressive subtype. It is characterized by loss of differentiation markers, increased proliferative and necrotic tendencies, reduced radioiodine uptake, and markedly elevated therapeutic difficulty (50). The 5 year survival rate lies between those of differentiated and undifferentiated carcinomas but remains substantially lower than that of typical PTC (2, 51). Molecularly, PDTC features driver gene alterations such as *TERT* promoter and *TP53* mutations, as well as aberrations in the *PI3K*/*AKT* signaling pathway, accompanied by upregulation of dedifferentiation markers in its transcriptional profile (52). Within the immune microenvironment, PDTC displays markedly reduced tumor infiltrating immune cells, including sparse T cells and TAMs, consistent with a “cold tumor/immune exclusion” phenotype (53). Expression of inhibitory immune checkpoints—PD-L1, PD-L2, PD-1, LAG-3, and TIM-3—are generally limited (54). In certain cases, mild immune activation signals can be detected, suggesting low immunogenicity and a propensity to develop an immune evasive microenvironment (55). Moreover, *BRAF*-associated signaling may further contribute to immune suppression and reduced tumor immunogenicity (18, 56). Collectively, PDTC exhibits an “immune inert and suppressive” signature, representing a refractory subtype that poses a major challenge for immunotherapeutic strategies in thyroid cancer. Representative immune interactions within the TIME of PDTC are shown in **Figure 3**.

### 4.1 T cells: reduced and enhanced exhaustion

In PDTC, the overall infiltration rate of T cells is lower than in PTC, with a particularly pronounced reduction in effector CD8^+^ T cells (18). The remaining CD8^+^ T cells show decreased expression of maturation markers such as GZMB and PRF1, accompanied by reduced MHC I/II expression and an enhanced exhausted phenotype, as indicated by the upregulation of immune checkpoints including PD-1, LAG-3, TIM-3, and TIGIT (56, 57). In parallel, the proportions of Tregs and immunosuppressive myeloid cells are elevated, along with increased signaling of inhibitory cytokines IL-10 and TGF-β, which suppress the functions of CD8^+^ T cells and NK cells, collectively contributing to a more pronounced immunosuppressive microenvironment (58, 59).

### 4.2 TAMs: M2 polarization and reduced antigen presenting capacity

TAMs infiltration is lower in PDTC than in PTC, but these macrophages predominantly shift from an M1-like to an M2-like phenotype (60). Elevated expression of M2 markers such as CD163, MRC1, and IL-10 is observed, accompanied by enhanced crosstalk with Tregs (61). Through the secretion of chemokines such as CCL22, TAMs recruit Tregs or suppressive T cells, while ligand–receptor interactions with tumor cells further reinforce the immunosuppressive network (62). The expression of costimulatory and antigen presenting molecules (MHC II, CD86, CD80) is downregulated, impairing the ability of TAMs to activate T cells (63, 64). In contrast, the expression of proangiogenic and matrix remodeling factors such as VEGF and MMPs is elevated, whereas secretion of proinflammatory cytokines IL-1β and TNF-α is markedly reduced (38, 65, 66).

### 4.3 NK cells: sparse infiltration and enhanced suppression

NK cells infiltration is markedly reduced in PDTC, with the population predominantly composed of immature or semi-mature subsets expressing inhibitory receptors such as NKG2A, TIGIT, and KLRG1, whereas mature NK cells expressing high levels of cytotoxic molecules GZMB and PRF1 are significantly decreased (65, 67). Tumor cells and stromal components upregulate inhibitory ligands including HLA-E/HLA-G and PD-L1, thereby suppressing NK cells cytotoxicity (65). The suppressive influence of myeloid cells on NK cells activity is also strengthened, accompanied by reduced expression of IFN-γ and TNF-α within NK cells, diminished activation signals (IL-15 or IL-12), and inhibition by immunosuppressive cytokines TGF-β and IL-10, collectively leading to attenuated cytotoxic function (18, 43).

### 4.4 B cells: immune cooling and reduced antibody production

B cells infiltration is sparse in PDTC, with decreased numbers of activated and plasmablast-like B cells, accompanied by reduced secretion of IgG and IgA (43, 68). The cooperative interaction between Tfh and B cells is weakened, and antigen presenting capacity is limited (69). Meanwhile, the expression of checkpoint molecules such as PD-1 is increased, further enhancing suppression of T cells activity (14, 69). The release of antitumor cytokines is diminished, whereas regulatory cytokines such as IL-10 are elevated, collectively driving an immunotolerant “silent state” within the tumor microenvironment (70, 71).

### 4.5 DCs: impaired antigen presentation and maturation barrier

A decline in the proportion of cDC1 (CLEC9A^+^, XCR1^+^) cells is frequently observed in PDTC (72). Tumor derived secretions and metabolic products inhibit DCs maturation, leading to downregulation of maturation markers CD80, CD86, and IL-12 (54). Consequently, DC–T cells costimulatory signaling (CD80/CD86–CD28) is disrupted, while DCs engage in inhibitory interactions with TAMs and tumor cells (70, 72, 73). The secretion of IL-12 and IFN-α/β is diminished, whereas IL-10, TGF-β, and suppressive chemokines such as CCL17 and CCL22 are upregulated, collectively resulting in defective activation of T helper and CD8^+^ T cells (74–76).

## 5 TIME of ATC

**Figure 4:**
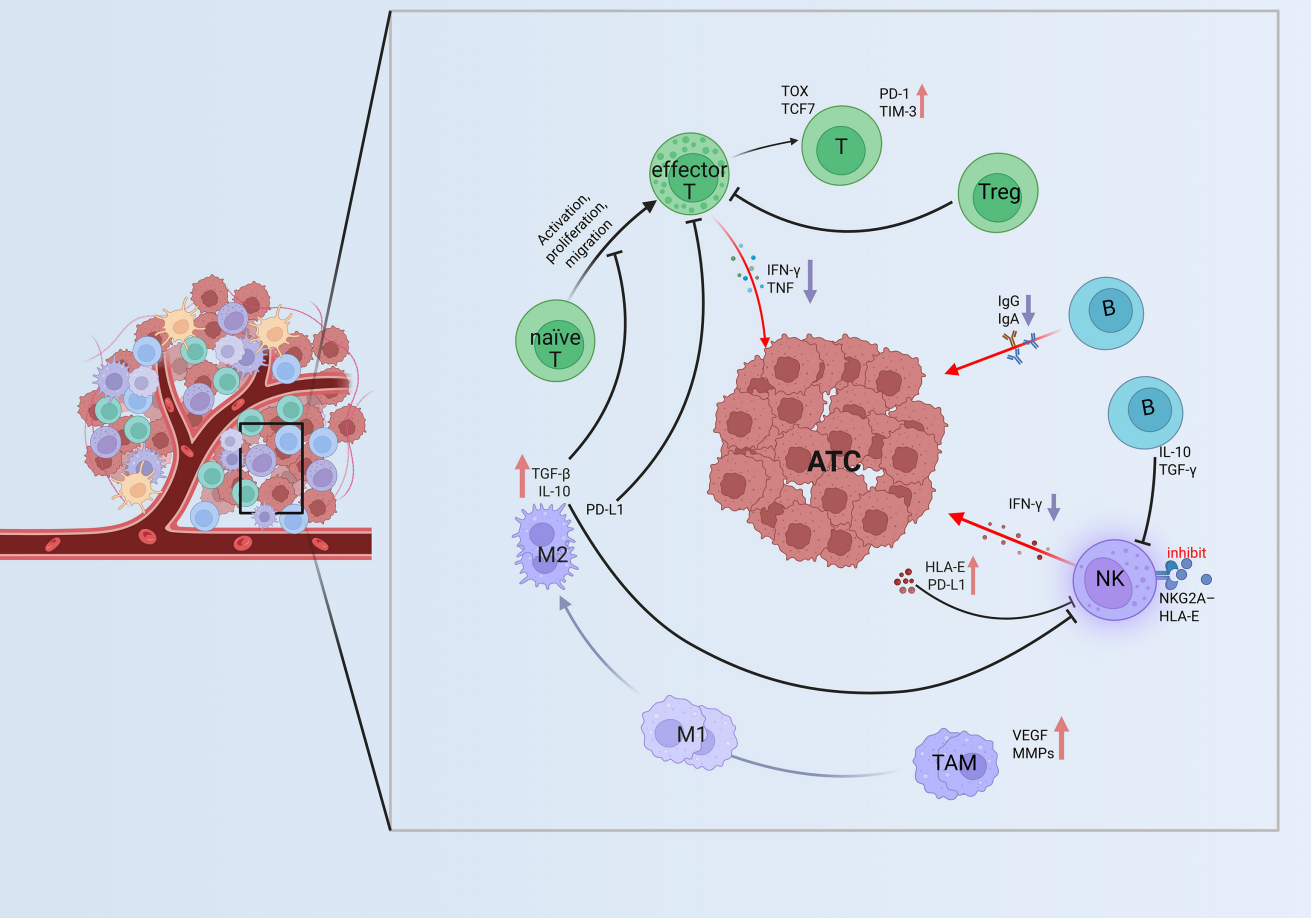
Immune dysfunction in the TIME of ATC. ATC features severe immune exhaustion, with effector T cells expressing PD-1, TIM-3, and TOX and producing less IFN-γ and TNF. Abundant Tregs and dominant M2 macrophages secrete TGF-β, IL-10, and PD-L1, reinforcing suppression. NK cells lose cytotoxicity via NKG2A–HLA-E signaling, creating a profoundly immunosuppressive TIME. ↑Purple downward arrows indicate decreased expression; ↓red upward arrows indicate increased expression. ATC, anaplastic thyroid carcinoma; naïve T, naïve T cell; effector T, effector T cell; T, Exhausted T cell; Treg, Regulatory T cell; B, B cell; NK, NK cell; M1, M1 type Tumor-Associated Macrophage; M2, M2 type Tumor-Associated Macrophage; TAM, Tumor-Associated Macrophages; TIME, tumor immune microenvironment; HLA-E, Human Leukocyte Antigen–E; VEGF, Vascular Endothelial Growth Factor; MMPs, Matrix Metalloproteinases; TOX, Thymocyte Selection–Associated High Mobility Group Box Protein; TCF7, Transcription Factor 7; TIM-3, T-cell Immunoglobulin and Mucin-domain Containing-3.

ATC represents one of the most aggressive forms of thyroid malignancy, characterized by rapid clinical progression, high resistance to conventional radioiodine therapy and most systemic treatments, and an extremely high mortality rate (19, 34, 77). ATC exhibits extensive immune cells infiltration accompanied by potent immunosuppressive signaling, which may partially explain its relatively higher clinical responsiveness to immune checkpoint blockade therapy (12, 34, 78). Representative immune dysfunctions within the TIME of ATC are shown in **Figure 4**.

### 5.1 T cells: high infiltration with “pre-exhausted/exhausted”

Multiple transcriptional profiling studies of ATC samples demonstrate that the overall infiltration of T cells—including CD8^+^, CD4^+^, and FOXP3^+^ Tregs—is higher than in most PTC specimens, yet their functional states are highly heterogeneous (15, 79, 80). One CD8^+^ T cells subset expresses cytotoxic molecules (GZMB, PRF1) indicative of effector activity, whereas another subset shows upregulation of exhaustion markers PD-1, LAG3, TIGIT, and TIM-3, along with lineage defining transcription factors TOX and TCF7, characteristic of pre-exhausted or terminally exhausted states (81–83).Trajectory analyses suggest that CD8^+^ T cells transition along an effector → pre-exhausted → terminally exhausted continuum, with enrichment of pre-exhausted populations that may represent potential targets for immune checkpoint blockade (15, 82).

Cell–cell communication analyses have identified several key inhibitory axes—PD-L1–PD-1, LGALS9–TIM-3, and CEACAM1–TIM-3—which are notably more active in ATC, indicating extensive local immunosuppressive signaling (34, 80, 84, 85). As exhaustion deepens, secretion of proinflammatory cytokines IFN-γ and TNF markedly declines, while immunosuppressive mediators released by TAMs and Tregs, such as IL-10 and TGF-β, further restrain effector T cells function, impair antigen presentation and cytotoxic responses, and undermine the stability of immunotherapeutic efficacy (86, 87).

The profound T cells exhaustion in ATC mechanistically supports PD-1 inhibitor activity, as seen in the spartalizumab trial where responses clustered in PD-L1^+^ patients (88). Yet, the low overall response rate (19%) and failures even in PD-L1 high cases confirm that PD-1 blockade alone cannot reverse this entrenched dysfunction, underscoring the need for combination strategies to effectively reinvigorate T cells.

### 5.2 TAMs: immune evasion and stromal remodeling

TAMs are markedly enriched in ATC. Single-cell transcriptomic analyses reveal that TAMs exist along a continuous polarization spectrum rather than a rigid “M1/M2” dichotomy, frequently exhibiting transcriptional programs associated with immunosuppression and tissue repair/fibrosis, characterized by high expression of CD163, MRC1, MARCO, APOE, TREM2, VSIG4, as well as SPP1 and MMPs (87, 89, 90). Studies have further demonstrated that TAMs harboring immunosuppressive gene signatures are significantly enriched in ATC and correlate with poor clinical outcomes (78, 79, 89).

TAMs interact intensively with tumor and lymphoid cells, expressing high levels of CCL2, CCL18, and CCL22 to recruit suppressive Tregs, while PD-L1 and IDO1 expression directly inhibits T cells effector function (91). Together with fibroblasts, TAMs co-secrete MMP9 and TGF-β, promoting tumor invasion and immune shielding (86, 89, 92). These macrophages often accumulate at the tumor–stroma interface, forming an “immunosuppressive belt” (79, 87). Elevated IL-10, TGF-β, and VEGF not only suppress cytotoxic immunity but also drive angiogenesis and fibrosis, thereby reprogramming the TIME and further dampening antitumor immune responses (93). Collectively, these cellular and molecular alterations reflect the profound invasiveness and immunological complexity of ATC (91, 94).

The myeloid-suppressive axis revealed by scRNA-seq explains primary resistance to PD-1 blockade in ATC (95). TAMs sustain T-cell exhaustion and promote Treg recruitment via alternative checkpoints and suppressive mediators (e.g., IL-10, TGF-β), creating a barrier insurmountable by PD-1 inhibition alone. This validates targeting the myeloid compartment to overcome resistance.

### 5.3 NK cells: functional blockade and suppression

In ATC, NK cells can be categorized into two main clusters: a mature effector subset (GZMB^+^/PRF1^+^) and an inhibitory subset characterized by high expression of NKG2A/KLRC1, TIGIT, and LAG3 (34, 96). Compared with PTC, the inhibitory subset is more dominant, and in certain cases, NK cells maturation or activation is markedly impaired (79). Tumor cells upregulate inhibitory ligands such as HLA-E, HLA-G, and PD-L1, which interact with NKG2A, KIRs, and PD-1, leading to profound suppression of NK cells cytotoxicity (12, 97). Moreover, M2 and Tregs further inhibit NK cells activity through secretion of TGF-β, IL-10, adenosine, and other metabolic mediators (86, 98). Spatial profiling reveals that NK cells preferentially localize at the tumor periphery or in perivascular niches rather than within the tumor core, forming so called “functionally isolated” or “immune cold” zones (99). Secretion of IFN-γ is markedly suppressed, resulting in diminished activation of antigen-presenting cells and restricted cross presentation, thereby weakening subsequent T cells responses and cooperative antitumor immunity (96, 98). Several studies have proposed strategies to reinvigorate NK cells function by blocking NKG2A or TIGIT, restoring IL-15 signaling, or combining STING (Stimulator of interferon genes)/TLR agonists to enhance cytotoxic reactivation (12, 100, 101).

### 5.4 B cells: pro-inflammatory and suppressive states

Within ATC tumors, B cells are observed across multiple maturation stages, including naïve (MS4A1^+^), activated, and plasmablast/plasma-cell–like lineages (XBP1^+^, MZB1^+^) (83, 102, 103). In certain lesions, tertiary lymphoid structures (TLSs) are formed, supporting local antibody production and Tfh–B cells interactions, which are associated with enhanced sensitivity to immunotherapy (104, 105). However, other B cells subsets exhibit marked upregulation of IL-10 and PD-1, reflecting the dual roles of B cells in antitumor immunity and immune tolerance, thereby shaping a complex immunological equilibrium (106–108). Spatial transcriptomic data further reveal that TLSs containing lesions often harbor both effector T cells and immunosuppressive populations, indicating the coexistence of local immune activation and restraint (103, 106). Functionally, B cells contribute to antibody dependent cellular cytotoxicity or complement dependent cytolysis, whereas regulatory B cells secrete IL-10 and TGF-β, directly suppressing T/NK cells activity and promoting immune tolerance (106–108).

### 5.5 DCs: cDC1 deficiency and restricted antigen presentation

In ATC, the absolute or relative loss of cDC1 (XCR1^+^, CLEC9A^+^) has been clearly identified as a hallmark of immune evasion (83, 109). Although cDC2 (CD1C^+^) subsets remain present, their maturation and IL-12 production are markedly impaired, with downregulation of CD80/86 and IL12A, leading to insufficient cross presentation and inadequate T cells priming (9, 110). Disruption of antigen presenting mechanisms results in widespread T cells exhaustion and immune unresponsiveness (12, 34). Costimulatory signaling via the CD80/86–CD28 axis is suppressed, while inhibitory pathways such as PD-L1/PD-1 and IDO1 become dominant, thereby hindering antigen specific T cells expansion (109, 111, 112). Spatial transcriptomic analyses reveal that functionally impaired DCs predominantly localize within tumor cores, whereas relatively intact DCs are enriched at the tumor margins and within TLSs regions (12, 113). Type I interferon (IFN-I) responses display marked heterogeneity, while IL-10 and TGF-β are enriched, further dampening effector T cells activation (110, 112, 113). Multiple studies have proposed that restoring DCs functionality through TLR agonists, STING pathway activators, or CD40 agonistic antibodies could enhance antitumor immune responses (111, 114, 115).

## 6 Comparison of the TIME differentiation of TC

**Figure 5:**
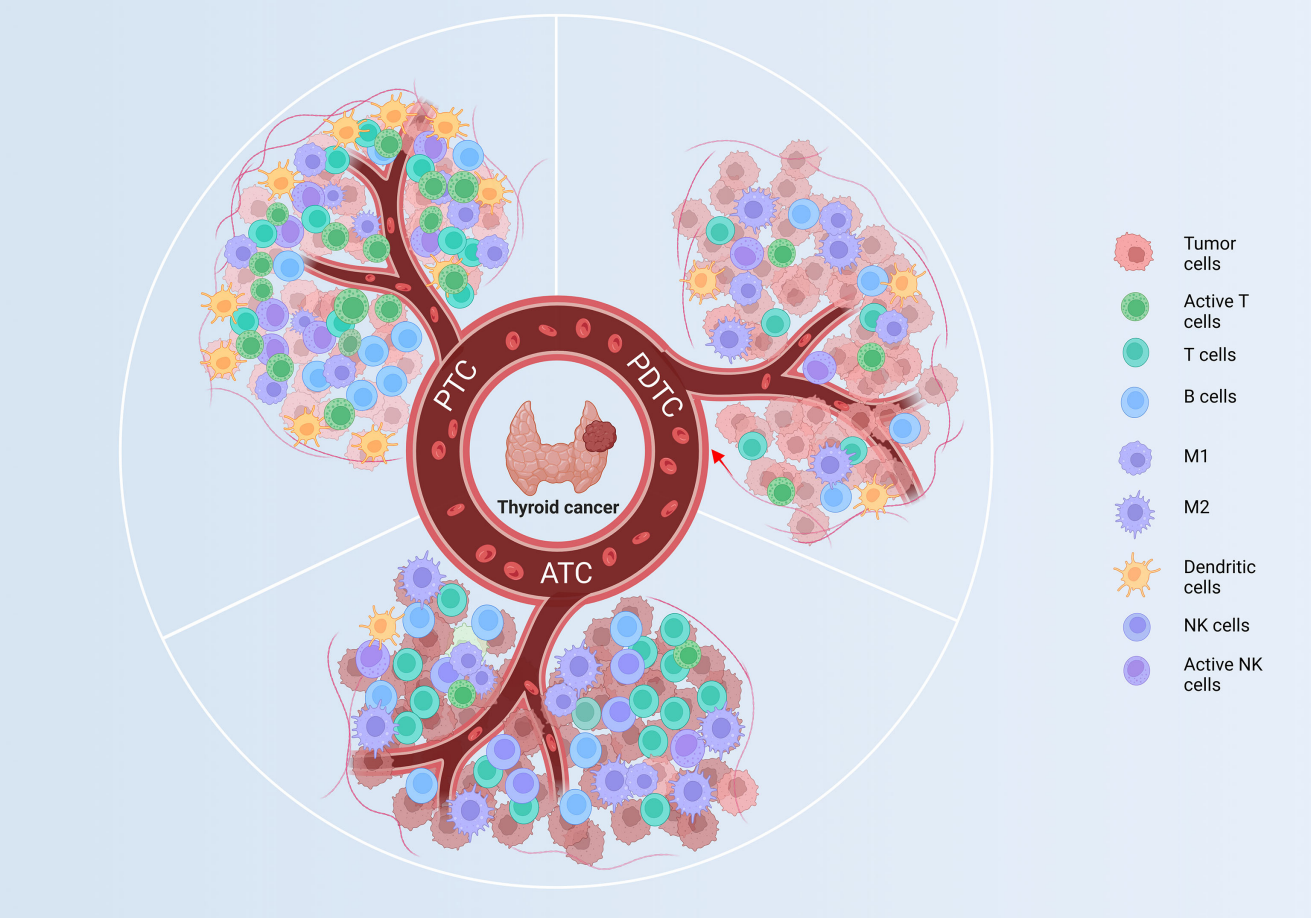
TIME evolution across thyroid cancer differentiation. This summary illustrates progressive immune remodeling from PTC to PDTC to ATC. PTC, “coexisting activation and suppression”, retains immune activation with active T cells and DCs; PDTC, “low infiltration/exclusion”, develops partial exhaustion and M2 polarization; ATC, “high infiltration with exhaustion”, shows complete suppression with exhausted T/NK cells and reduced antigen presentation. ATC, anaplastic thyroid carcinoma; PTC, papillary thyroid carcinoma; PDTC, poorly differentiated thyroid carcinoma; TIME, tumor immune microenvironment; DC, Dendritic cell; M1, M1 type Tumor-Associated Macrophage; M2, M2 type Tumor-Associated Macrophage.

As the degree of differentiation decreases, the TIME of TC exhibits a progressive transition from “coexisting immune activation and suppression” → to a “cold/immune excluded” state → and finally to a “highly infiltrated but exhausted” phenotype (9, 12, 34, 79). This gradient is accompanied by increasing heterogeneity, dedifferentiation, and therapeutic refractoriness. The dynamic remodeling of the TIME across thyroid cancer differentiation is illustrated in **Figure 5**.

Characterized by a “coexistence of immune activation and suppression” state, with moderate immune cells infiltration and partial preservation of cytotoxic CD8^+^ T cells function (32). Immune evasion primarily depends on CD8^+^ T cells exhaustion and the immunosuppressive network orchestrated by M2-polarized TAMs and Tregs (86, 116). This immune landscape correlates with increased tumor aggressiveness and radioiodine (RAI) refractoriness, suggesting the potential of strategies that enhance immunogenicity and promote antigen presentation or redifferentiation reactivation.

Displays a “cold tumor/immune excluded” phenotype, featuring impaired CD8^+^ T cells maturation, TAMs mediated immunosuppressive remodeling, reduced and dysfunctional NK cells activity, attenuated Tfh–B cells cooperation, increased PD-L1 expression, decreased cDC1 frequency, and defective antigen presentation (56, 59, 61, 74, 75, 117). This subtype represents a major immunotherapeutic challenge, strongly associated with RAI resistance and treatment failure. Therapeutic strategies targeting this immune profile may include myeloid reprogramming combined with dual/multiple checkpoint blockade and restoration of antigen presentation capacity.

Exhibits a “highly infiltrated yet exhausted” immune state, characterized by extensive TIME reprogramming, high level expression of multiple checkpoint pathways (PD-1/PD-L1, LAG-3, TIM-3, etc.), and massive enrichment of myeloid derived suppressor cells (118, 119). Inhibitory NK cells dominate, while TLSs formation and upregulation of IL-10 and PD-L1 reflect the functional duality of B cells. Absolute or relative loss of cDC1 and impaired maturation of cDC2 lead to disrupted antigen presentation, spatially forming an “immunosuppressive loop” and a functionally compromised tumor core (9, 18, 78, 79). Although ATC retains partial responsiveness to immune checkpoint inhibitors, its therapeutic benefit is highly heterogeneous. Optimal strategies may require reversal of T cells exhaustion, remodeling of myeloid suppression, and restoration of cDC1 functionality to maximize clinical efficacy.

The profound immune dysfunction and heterogeneity observed in ATC may be rooted in its cellular origins. The prevailing model posits that ATC arises through the dedifferentiation of pre-existing differentiated carcinomas. Genomic analyses delineated this trajectory, identifying key co-mutations (e.g., TERT and TP53) that drive progression (119). Crucially, single-cell transcriptomics visually captured this process, revealing ‘anaplastic-like’ cells residing within BRAF-mutant papillary thyroid carcinomas, thereby providing direct evidence of clonal evolution along a dedifferentiation continuum (34).

## 7 The applications of spatial transcriptomics in the TIME of TC

While scRNA-seq excels at cataloging cellular diversity, it lacks spatial context. Spatial transcriptomics directly addresses this by mapping gene expression within intact tissue architecture, providing critical insights into the thyroid cancer ecosystem. This technology validates cell-cell interactions predicted by scRNA-seq and reveals key spatial patterns of immune evasion. For instance, it has visually confirmed the presence of tertiary lymphoid structures in PTC, localizing active immune collaborations. In contrast, it delineates the “immune-excluded” phenotype in PDTC and ATC, demonstrating how cytotoxic CD8^+^ T cells are restricted to the tumor stroma or invasive margin, while the core is dominated by immunosuppressive cells like M2-polarized TAMs. The co-localization of exhausted T cells with PD-L1^+^ myeloid cells provides a spatial rationale for immunotherapy responses. Ultimately, the synergy between scRNA-seq (identifying the “players”) and spatial transcriptomics (defining the “stage”) is forging a spatially-resolved understanding of the TIME, which holds promise for guiding spatially-informed precision immunotherapy in the future.

## 8 Discussion

Advances in Single-Cell and Spatial Omics Are Driving a Paradigm Shift in TIME Research—from Quantitative Assessment to Qualitative Understanding. The evolution of analytical approaches—from quantifying immune cells infiltration density and immune checkpoint expression (e.g., PD-1, PD-L1) (18, 120), to single-cell and spatial transcriptomic mapping that delineates lineage trajectories, functional reprogramming, and intricate cell–cell communication networks (9, 78, 118)—has elevated the focus from “immune cells quantity” to the broader concept of “immune ecology.” These advances have revealed a strong correlation between tumor differentiation status and the dynamic evolution of TIME in thyroid carcinoma (86, 116). The dedifferentiation continuum from PTC → PDTC → ATC is mirrored by TIME transitioning from “coexistent activation and suppression” to “cold/immune excluded” and ultimately to a “highly infiltrated but exhausted” state. This progressive pattern provides a biological explanation for clinical heterogeneity and reflects the bidirectional co-evolution between tumor cells and their immune microenvironment.

For studying dedifferentiation and TIME, future directions evolution include (1): multi-omics integration and spatial reconstruction (121), enabling dynamic tracking of key cells populations—such as pre-exhausted CD8^+^ T cells, TREM2^+^ TAMs, and cDC1s—before and after therapy (2). Single-cell trajectory analyses to monitor lineage transitions and functional state shifts during tumor progression (122) (3). Spatial transcriptomics to identify the precise localization of critical immune populations, constructing a three-dimensional immune ecosystem and providing spatially resolved targets for disrupting immunosuppressive networks (123, 124). From a therapeutic perspective, mechanism driven combination strategies should be tailored according to differentiation hierarchy: DTC: immune activation approaches based on antigen presentation and T cells restoration (such as cancer vaccines or local radiotherapy combined with PD-1 blockade) (125, 126); PDTC: myeloid reprogramming combined with multi-checkpoint inhibition (53); ATC: highest potential for combinatorial immunotherapy, emphasizing anti-exhaustion strategies, STING pathway activation, and stromal unlocking NK cells reactivation (127–129). Recently, targeted plus immunotherapy neoadjuvant regimens—for example, *BRAF^V600E^* driven combinations—have entered clinical trials and yielded promising early results, demonstrating the feasibility of achieving both rapid tumor regression and durable immune responses within a short therapeutic window (130). Key clinical studies targeting the tumor immune microenvironment are summarized in **Table 3**.

**Table 3:** Therapeutic applications targeting the tumor immune microenvironment in thyroid cancer.

Types	Study types	Design(n)	Intervention	Targets	Efficacy	Limitations	References
ATC	Interventional	42	Spartalizumab	PD-1	Efficacy correlated with PD-1^+^ and CD8^+^ T cells infiltration	No control group, long-term adverse effects unassessed	Capdevila J, et al., 2020 (88)
ATC	Interventional	42	Atezolizumab + Targeted therapy	PD-L1 *BRAF^V600E^* *RAS*	Significant therapeutic efficacy	Lack of placebo control inconsistent treatment duration	Cabanillas ME, et al., 2024 (131)
PTC	Interventional	57	Pembrolizumab + Lenvatinib	PD-L1	Pembrolizumab improved efficacy by increased PD-1^+^ CD8^+^ T cells infiltration	Non-randomized controlled design	French JD, et al., 2024 (132)
PTC	Interventional	10	Surufatinib + Toripalimab	CSF-1R PD-1	Marked therapeutic efficacy	High incidence of adverse events Limited sample size	Chen J-y, et al., 2023 (133)
TC	Observational	10	Lenvatinib	VEGFR1–3 PDGFRα	Increased proportion of NK cells	Limited sample size, no histological subtypes stratification	Jin M, et al., 2023 (134)
ATC/ PDTC	Observational	6 ATC 2 PDTC	Pembrolizumab Lenvatinib	PD-L1	Marked therapeutic benefit	Limited sample size, high rate of grade III/IV toxicities	Dierks C, et al., 2021 (135)

Recent clinical trials highlight promising efficacy of PD-1/PD-L1–based immunotherapy, particularly in combination with targeted agents, across thyroid cancer subtypes, yet therapeutic benefit remains constrained by immune-related toxicity, limited sample sizes, and lack of randomized control. ATC, anaplastic thyroid carcinoma; PTC, papillary thyroid carcinoma; PDTC, poorly differentiated thyroid carcinoma; VEGFR, Vascular Endothelial Growth Factor Receptor; PDGFR, Platelet Derived Growth Factor Receptor; CSF-1R, Colony Stimulating Factor 1 Receptor; Retrospective, Retrospective Clinical Study; Prospective, Prospective Clinical Study.

The single-cell architecture of the ATC TIME elucidates the limited efficacy of PD-1 blockade, revealing that PD-L1 upregulation occurs within a profoundly immunosuppressive ecosystem. This resistance is multifaceted: CD8^+^ T cells are locked in pre-exhausted or terminally exhausted states; M2-polarized and TREM2^+^ TAMs dominate, secreting IL-10, TGF-β, and adenosine; and loss of cDC1 with impaired cDC2 maturation creates an antigen presentation deficit (88, 95). Consequently, PD-1 monotherapy often fails to reverse this entrenched suppression. In contrast, the success of dabrafenib plus trametinib in BRAF-mutant ATC demonstrates that targeted therapy can achieve profound tumor regression (136), while emerging evidence suggests MAPK inhibition may also favorably remodel the TIME. This synergy is exemplified by lenvatinib plus pembrolizumab, pointing toward a future paradigm of rational combinations—co-targeting tumor cells, specific immune resistance hubs (e.g., TREM2^+^ TAMs), and checkpoint pathways—to overcome resistance.

This review primarily reflects the immune landscape of PTC, PDTC, and ATC. ScRNA-seq data for FTC and MTC remain limited, largely due to their lower incidence and the challenges in procuring fresh surgical specimens. Existing studies are often constrained by small sample sizes and potential selection biases. Future multi-center collaborations are essential to define the TIME of these understudied subtypes.

In summary, high resolution temporal and spatial immune mapping has delineated an evolutionary TIME atlas from differentiated to undifferentiated thyroid carcinoma, laying a conceptual and technological foundation for precision immunotherapy. This paradigm shift—from static immune cells distribution to dynamic immune system reconstruction—highlights a state stratified, mechanism guided framework for adaptive clinical trial design and marks the beginning of a mechanistically driven era in thyroid cancer immunotherapy.

## Glossary

ATC: Anaplastic thyroid carcinoma
APOE: Apolipoprotein E
BRAF: v-Raf murine sarcoma viral oncogene homolog B
CD8⁺: CD8 positive T cells
CTLA-4: Cytotoxic T lymphocyte–associated protein 4
CD4⁺: CD4 positive T cells
cDC2: Conventional dendritic cell type 2
cDC1: Conventional dendritic cell type 1
DCs: Dendritic cells
DTC: Differentiated thyroid carcinoma
FTC: Follicular thyroid carcinoma
GZMB: Granzyme B
HAVCR2: Hepatitis A virus cellular receptor 2
IFN-γ: Interferon gamma
IDO1: Indoleamine 2, 3-dioxygenase 1
IL: Interleukin
LAG3: Lymphocyte activation gene 3
MMPs: Matrix metalloproteinase
MRC1: Mannose Receptor C Type 1
MDSC: Myeloid derived suppressor cells
MARCO: Macrophage Receptor with Collagenous Structure
MHC: Major histocompatibility complex
NK: Natural killer cells
PRF1: Perforin-1
PTC: Papillary thyroid carcinoma
PDTC: Poorly differentiated thyroid carcinoma
PD-1: Programmed cell death protein 1
PD-L1: Programmed death ligand 1
RAI: Radioactive iodine
STING: Stimulator of interferon genes
SPP1: Secreted Phosphoprotein 1
scRNA-seq: Single-cell RNA sequencing
TIME: Tumor immune microenvironment
Tregs: Regulatory T cells
TAMs: Tumor-associated macrophages
Th: Helper T cells
Tfh: Follicular helper T cells
TREM: Triggering Receptor Expressed on Myeloid Cells 2
TC: Thyroid carcinoma
TIGIT: T cell immunoreceptor with IG and ITIM domains
TGF-β: Transforming growth factor beta
TNF-α: Tumor necrosis factor alpha
TLSs: Tertiary lymphoid structures
TCF7: Transcription factor 7
TOX: Thymocyte selection associated high mobility group box protein
TIM-3: T-cell immunoglobulin and mucin-domain containing-3
TLR: Toll-like receptor
VSIG4: V-Set and Immunoglobulin Domain Containing 4
VEGF: Vascular endothelial growth factor.
